# A Six-Year Prospective Comparative Study of Wide and Standard Diameter Implants in the Maxillary and Mandibular Posterior Area

**DOI:** 10.3390/medicina57101009

**Published:** 2021-09-25

**Authors:** Puneet Wadhwa, Seung-Kook Kim, Hyun-Jin Kim, Ho-Kyung Lim, Qi Jia, Heng-Bo Jiang, Eui-Seok Lee

**Affiliations:** 1 Department of Oral and Maxillofacial Surgery, Korea University Graduate School of Clinical Dentistry, Seoul 08308, Korea; puneet@korea.ac.kr (P.W.); openmind25@naver.com (S.-K.K.); kim-dentist@hanmail.net (H.-J.K.); ungassi@naver.com (H.-K.L.); 2 The Conversationalist Club, School of Stomatology, Shandong First Medical University & Shandong Academy of Medical Sciences, Tai’an 271016, China; aqms-happy@outlook.com (Q.J.); hengbojiang@hotmail.com (H.-B.J.)

**Keywords:** wide diameter implants, standard diameter implants, survival rate, marginal bone level

## Abstract

*Background and Objectives*: The aim of our study was to test whether wide diameter (6 mm) implants perform differently from standard diameter (4 mm) implants in terms of marginal bone level and survival rate. *Materials and Methods*: Our sample comprised 72 patients who underwent surgery; a total of 80 implants were placed in the maxillary or mandibular molar region. Patients were divided into two groups according to the diameter of the implant, and were followed up for six years after the final setting of the prosthetics. In the test group, 40 implants with 6-mm diameter were inserted; in the control group, 40 standard diameter implants were inserted. Using panoramic radiographs, we investigated mesial and distal marginal bone levels around the implant fixtures. *Results*: After the first implant surgery, three implants, including one wide diameter and two standard diameter implants, failed due to lack of osseointegration. We did not note any fixture fracture during the six-year follow-up. After loading, we observed a six-year survival rate of 97.29% with no statistically significant difference from standard diameter implants, with a survival rate of 94.87%. *Conclusions*: This study shows that 6-mm diameter implants may be considered in the presence of adequate alveolar ridge width in the posterior maxillary and mandibular regions.

## 1. Introduction

After tooth loss, dental implant placement is one method of tooth restoration. Since Brånemark established the concept of osseointegration, surgeons have been using a variety of implant types and sizes depending on the shape of the alveolar bone [1]. Implant placement has proved to be beneficial in reducing bone loss after tooth extraction [2]. After tooth loss, bone resorption is inevitable, limiting the selection of implant diameter. Surgeons have used implants that are wider than standard implants to improve initial implant fixation in patients with low alveolar bone density [3]. An increase in implant diameter has been shown to improve implant stability at the time of placement [4]. Initially, implants with diameters greater than 6 mm were used primarily for the re-implantation of failed standard-diameter implants or for immediate placement after tooth extraction to obtain adequate initial fixation [5,6,7]. In addition, wider diameter implants are superior to different diameter implants in terms of biomechanical osseointegration, primary stability, and stress-dispersing ability [8].

However, the possibility of marginal bone loss increases with the increase in implant diameter due to excessive pressure on the buccal bone. This results in gingival recession; therefore, the routine use of wide-diameter implants is controversial [9,10]. Some studies have suggested increased marginal bone loss with an increase in diameter [11]. In a study, wide diameter implants (8 or 9 mm), when used for the restoration of single mandibular molar teeth, led to a relatively high rate of failure after one year [12]. However, in more recent studies, the use of wide diameter implants has shown satisfactory results, even after immediate placement following molar extraction after one-year follow-up [13,14]. The use of a short, wide diameter implant has been shown to have no adverse effect on survival [15]. Short-term follow-up studies have shown high survival rates after placement of 5- and 6-mm diameter implants for up to two years [16].

There are many long-term studies for evaluating the survival rate and marginal bone levels of dental implants [17,18,19], but very few studies have examined the long-term survival rate for wide diameter implants. The purpose of this prospective study was to evaluate long-term clinical prognoses of wide diameter (6 mm) implants in comparison with standard diameter implants (4 mm).

## 2. Materials and Methods

### 2.1. Study Design and Study Population

He we report on a nonrandomized, single-center prospective study conducted in the Oral and Maxillofacial Surgery Department, Graduate School of Clinical Dentistry, Korea University Guro Hospital. This study was approved by the Institutional Review Board (IRB No.: MD 10023) of our institution and conducted according to the principles of the Declaration of Helsinki. All patients agreed to participate in the study and signed the consent form.

In this study, we enrolled 72 patients who required one or more tooth restoration in the posterior region and who visited our dental clinic in 2013 and 2014. A total of 80 implants were placed, 40 in each group: wide diameter (6 mm) implant group (experimental group) and standard diameter (4 mm) implant group (control group). Our selection criteria were as follows: (i) patients who were over 18 years of age and needed implants because of tooth loss; (ii) patients who had sufficient buccolingual and mesiodistal alveolar bone for implant insertion; (iii) sinus floor augmentation but no primary bone augmentation procedures; (iv) nonsmokers; and (v) patients who agreed to participate in the clinical study and signed the consent form. Exclusion criteria were as follows: (i) patients with uncontrolled systemic disease; (ii) pregnant women; (iii) patients with hypersensitivity to implants; and (iv) patients who were considered inappropriate for participation in other clinical trials. We limited implantation to the maxillary or mandibular molar area and defined four implant lengths, i.e., 7 mm, 8.5 mm, 10 mm, and 11.5 mm, based on the distance from the main anatomical structure, such as the intra-alveolar canal and maxillary sinus.

### 2.2. Surgical Methods

A single experienced surgeon placed all the implant fixtures in the maxilla or mandible. Patients were given antibiotics and nonsteroidal analgesics/antiphlogistics before surgery. A crestal incision was made, and a full-thickness flap was raised under local anesthesia. If a sufficient amount of bone required for implant placement was not present, the patient was not included in the study, and a different treatment plan was offered in accordance with clinical protocols. Holes were drilled into the bone to place titanium dental implant fixtures. In the test group, 40 wide implants with 6-mm diameter (Osstem TSIII Ultra-Wide Fixture, Seoul, Korea) were inserted, whereas in the control group, 40 standard diameter implants with 4-mm diameter (Osstem TSIII Standard Fixture, Seoul, Korea) were inserted. If needed, Bio-Oss xenograft was used during surgery. After placing the fixture into the bone, the surgeon carefully sutured the opening. If the insertion torque was inadequate, the surgeon conducted the surgery in two stages for sufficient osseointegration, whereas if the torque was sufficient, the surgeon connected a healing abutment without a second surgery.

### 2.3. Measurement of Marginal Bone Levels and Implant Survival

**Figure 1:**
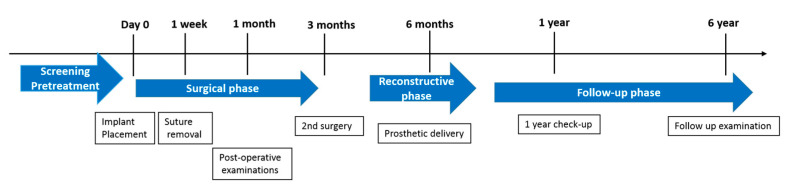
Time sequence of treatment and 6 year follow-up.

**Figure 2:**
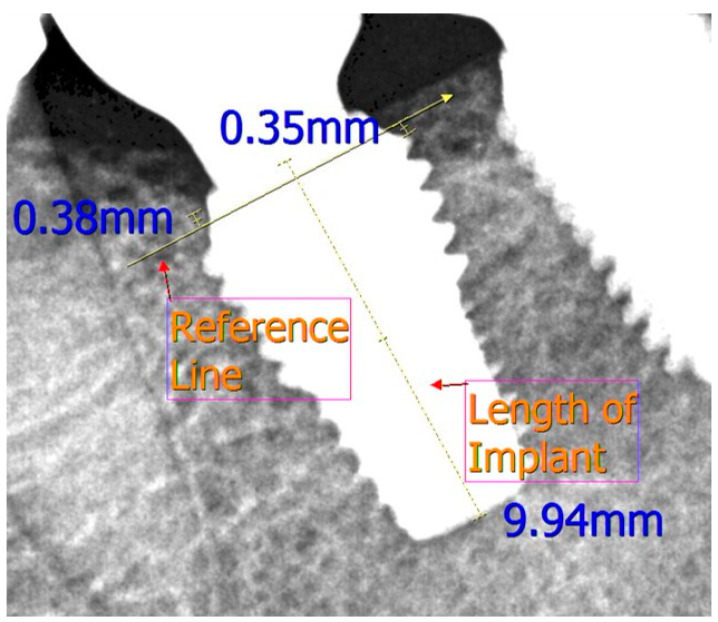
Measurement of the mesial and distal marginal bone level from the reference line in the test group.

We set a schedule of implant placement, removal of stitches, one-month checkup, second surgery, prosthetic appliance delivery, one-year follow-up, and six-year follow-up (Figure 1). At each visit, a digital panoramic radiograph was taken and the mesial and distal marginal bone levels around the fixtures were measured using a distance measurement program (Starpacs, Infinitt, Seoul, Korea); this procedure is similar to that used in a previous study [20]. Taking the magnification factor into account, we corrected the measured values using the length of the implant and thread pitch; the reference line was taken from the start of the rough surface of the implant (Figure 2). We expressed the measured values in millimeters and calculated the average values of marginal bone level in the mesial and distal surfaces. Analysis of the radiographs was done twice at a two-week interval by the same examiner who was blinded from the study.

The implant was considered a failure if it was lost or unstable (due to early failure to osseointegrate, late loss of osseointegration, or implant fracture). The implant survival rate was evaluated after six years. Implant survival was designated as a functional and stable implant with no clinical or radiological pathology.

### 2.4. Statistical Analyses

We performed all statistical analyses using IBM SPSS Statistics for Windows, version 19.0 (IBM Corporation, Armonk, NY, USA). First, a Shapiro Wilk normality test was performed. The Mann–Whitney U test was used for comparison of the marginal bone levels of the two groups because of the non-normality of the data. To compare the survival rate of implants between the two groups, we used Fisher’s exact test. A *p*-value less than 0.05 was considered statistically significant.

## 3. Results

The mean ages of patients included in this study were 52.83 and 54.27 years in the test and control groups, respectively. A total of 40 implants were placed in each group. Patient demographic data are given in Table 1. The total evaluation period was six years after prosthodontic treatment during which no patient experienced temporomandibular joint disorders. Three implants in the wide diameter group and one in the standard diameter group were not available at six-year follow-up, so their data was not used for survival rate analysis. The total number of implants in the wide and standard diameter groups was 37 and 39, respectively (Table 2).

### 3.1. Implant Survival Rates

**Figure 3:**
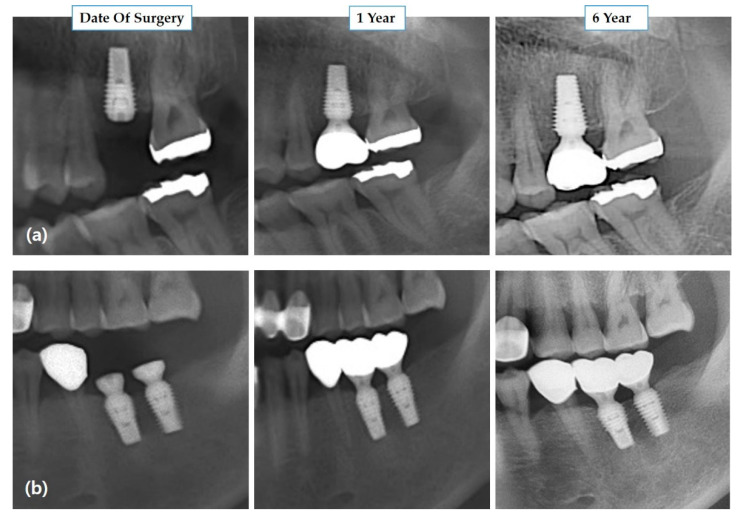
Radiologic evaluation of the wide diameter implant in two different patients. (**a**) Implant placement in the left maxillary posterior region on the day of implant placement, one year and six years after prosthetic loading. (**b**) Implant placement in the left mandibular posterior region on the day of implant placement, one year and six years after prosthetic loading.

Of the total implants inserted, osseointegration failed in three cases, i.e., one implant in maxilla from the wide diameter group and two implants placed in the mandible from the standard diameter group failed to osseointegrate. The fixture was dislocated two weeks after the primary implant surgery in the test group and about two months after surgery in the control group. After three months of healing, the patient again underwent implantation but was excluded from this study. During the six-year observation period, no other implant was lost in any group, resulting in six-year survival rate of 97.29% and 94.87% in both groups (Table 2). Figure 3 shows radiological images of two different patients with wide-diameter implants at different follow-up times. Fisher’s exact test showed an insignificant difference in the survival rate of the wide and standard diameter implants.

### 3.2. Marginal Bone Level

**Figure 4:**
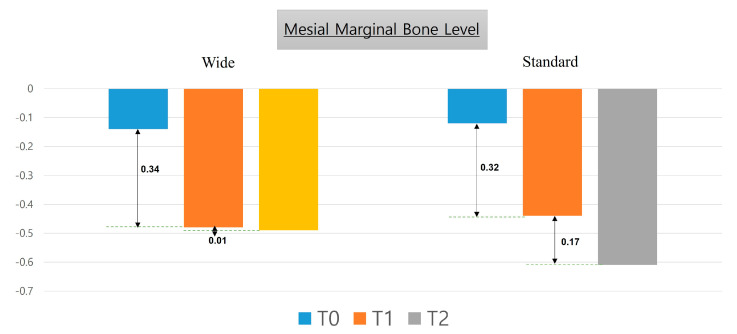
Changes in mesial marginal bone level six years after the placement of wide-diameter as well as standard-diameter implants.

**Figure 5:**
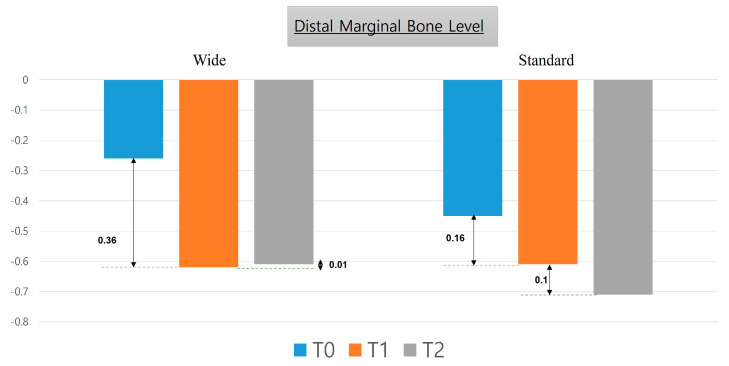
Changes in distal marginal bone level six years after the placement of wide-diameter as well as standard-diameter implants.

Table 3 describes the mesial and distal marginal bone level values from the time of implant placement up to six years after prosthetic rehabilitation. There was no statistically significant difference between the two groups. Figure 4 and Figure 5 describe changes in mesial and distal marginal bone levels, respectively, in graphical form.

## 4. Discussion

Since the late 1980s, wide-diameter implants have been used to improve primary implant stability in patients with poor supporting bone quality [3,21]. Although the application of these implants is becoming increasingly widespread, there are only a few reports on their use to date.

The effect of implant diameter on marginal bone loss and implant stability is controversial. Some studies have shown that implants with 5-mm or larger diameters may exert excessive pressure on the buccal bone, resulting in lower survival rates [22]. However, in this study, a marginal bone loss of 0.2 mm or more was not observed, even after the use of an implant with a 6-mm diameter for six years, and the six-year survival rate was also very high (97.29%). The survival rate in our study was better than the 76.3% survival rate of five years for a wide-platform, 5-mm diameter implant reported in an earlier study [23]. Our result is similar to another study with an approximately four-year survival rate of 98.28% for 6–7 mm diameter implants [24]. According to a previous study, the larger the diameter, the lower the stress on the cortical bone around the cervical portion of the implant during occlusion [25,26]. Wider diameter implants have also previously exhibited higher primary stability [27]. These findings support the use of wide diameter implants.

Although this study focuses on the effect of the diameter of the implant, some other factors may also affect the marginal bone levels. The incorporation of a cantilever can be thought to have an effect on the marginal bone level. However, in a study, it was shown to have no significant effect on the implant adjacent to the cantilever [28]. In long-term follow up, a higher clinical crown-to-implant ratio is associated with greater marginal bone loss in short dental implants in the mandibular posterior region [29]. Occlusal overload enforced on the prostheses can also contribute to marginal bone loss [30]. An off-axial loading is due to a disparity between the crown and implant width. If the mesiodistal width of the crown increases, the potential for off-axis loading also increases. However, no significant changes in marginal bone level were detected in the axial and off-axial loading of implants [31].

In this study, 36 out of 37 implants showed success after six years, similar to the findings from other studies. The six-year survival rate was high (97.29%). Some medium-term studies have reported satisfactory survival rates. Degidi and Piattelli [32] reported a survival rate of 97.2% for implants with a 5.5-mm or larger diameter, and some other studies have reported one-year survival rates of >95% [9,33]. Very similar survival rates have been reported for implants with all diameters, except wide-diameter implants [34,35,36,37]. Instead, some studies showed that survival rates are more closely related to the method of implant surface treatment and the initial stability of implant, rather than implant diameter [9,38].

The marginal bone level after implantation gradually decreased over time. The amount of change during the follow-up is depicted in the graph in Figure 4 and Figure 5. In addition, we observed negligible marginal bone loss after 12 months following prosthetic restoration; this result was similar to that from Ku et al. [24].

In this study, no implant fractures occurred during the follow-up period. In another study with standard-diameter implants (4 mm or less), the incidence of implant fracture was 1.4%, and the maximum fractures occurred within three to four years of implant placement [27]. In this study, one implant failed within one month of placement due to a lack of osseointegration. The failed implant was 8.5 mm in length and was placed in the maxillary posterior area approximately four months after tooth extraction.

Some studies have shown discouraging results for immediate placement of 6- to 8-mm implants when compared with ridge preservation and delayed placement [7]. In contrast, in our study, eight wide-diameter implants were immediately placed after extraction, all of which survived during the study period. Further studies with larger sample sizes are needed to corroborate our findings.

Peri-implantitis is diagnosed when there is inflamed mucosa along with positive bleeding on probing, periodontal depth ≥5 mm, and cumulative bone loss of ≥2 mm and/or ≥3 threads of implant are exposed [39]. In a study by Rinke et al., the prevalence of peri-implantitis was 11.2% [40]. In this study, four wide-diameter implants, i.e., three in the mandible and one in maxilla, showed peri-implantitis three years after implantation. One implant in the mandible was immediately placed after extraction. The implants were treated with implantoplasty, and they survived. Thus, the six-year success rate in our study was 86.1%. We determined the success rates of implants based on the method described by Smith and Zarb [41,42]. We could not find any other study on the survival and success rates of wide-diameter implants with such a long-term follow-up. In the standard diameter group, three implants showed peri-implantitis, of which two were placed in the maxilla with delayed placement and one was immediately placed after extraction in mandible. The most common technical complication in our study was the loosening of the abutment screw.

No implant fracture was observed in our study in any group, indicating that the placement of wide-diameter implants in the posterior regions may be beneficial, especially in patients with parafunctional habits. In the presence of adequate width of the alveolar ridge, wide-diameter implants may be suitable for immediate placement after tooth extraction or for the replacement of failed standard-diameter fixtures. In a study, when a wide-diameter implant was placed immediately after extraction of a molar, the marginal bone level loss was higher compared to that of the delayed placement group [13]. All wide diameter implants had the same surface treatment in this study. Digital panoramic radiography was used in this study, as a digital radiographic image provides a better assessment of the bone level in comparison to an analogue film. Although panoramic radiography is less sensitive than the periapical technique for measuring marginal bone levels, it requires the use of the bisector technique for the maxillary posterior region, which results in differences in angulation that can alter marginal bone level measurements [43].

Further studies with larger sample sizes are needed to corroborate our findings.

Wide-diameter implants do not pose any difficulties during insertion in the upper or the lower posterior parts of the mouth unless the patient lacks sufficient bone or there are systemic disease-causing complications.

In this study, wide-diameter (6 mm) implants showed an excellent six-year survival rate (97.29%) in comparison with standard diameter implants (94.87%) with reduced marginal bone loss during a six-year follow-up period.

## Figures and Tables

**Table 1:** Patient demographic data.

Implants		Wide n = 40	Standard n = 40
Anatomical location	Maxilla	22	19
	Mandible	18	21
Sex	Male	29	20
	Female	7	16
Age (years)	Mean	52.83	54.27
Immediately placed after extraction	Yes	8	4
	No	32	36
Bone graft (Bio-Oss)	Yes	10	17
	No	30	23
Surgical Technique	One-Stage	14	11
	Two-Stage	26	29
Sinus Floor Elevation	Yes	4	4
Implant Length	7 mm	2	2
	8.5 mm	3	13
	10 mm	26	18
	11 mm	9	7

**Table 2:** Six-year survival and failure rate.

Group	Total Implants	Lost To Follow-Up	Implants Lost	Implants Survived	Survival Rate (%)	Failure Rate (%)	Fisher’s Exact Test
Wide	40	3	1	36	97.29	2.70	*p*-value > 0.05
Standard	40	1	2	37	94.87	5.13	

**Table 3:** Correlation between marginal bone level (MBL) and postoperative time. SD, standard deviation.

MBL		n	Mean ± SD	Median	Range	n	Mean ± SD	Median	Range	*p*-Value
Implant placement	Mesial	40	−0.14 ± 0.34	−0.93	−2.55 to 0	40	−0.12 ± 0.58	0	−1.01 to 1.24	0.8248
	Distal		−0.26 ± 0.49	−1.05	−2.25 to 0		−0.45 ± 0.33	−0.6	−1.46 to 0	0.9436
	Mean		−0.2 ± 0.52	−0.99	−2.23 to −0.44		−0.28 ± 0.35	−0.175	−1.235 to 0.35	0.5382
1 Year	Mesial	39	−0.48 ± 0.81	−0.54	−2.12 to 1.22	38	−0.44 ± 0.44	−0.425	−1.3 to 0.55	0.8018
	Distal		−0.62 ± 0.63	−0.62	−2.79 to 0.87		−0.61 ± 0.45	−0.585	−1.9 to 0.45	0.9764
	Mean		−0.55 ± 0.62	−0.64	−1.83 to 1.03		−0.52 ± 0.37	−0.5925	−1.2 to 0.375	0.8606
6 Years	Mesial	36	−0.49 ± 1.02	−0.4	−2.9 to 1	37	−0.61 ± 0.65	−0.54	−3.07 to 0.38	0.5507
	Distal		−0.61 ± 1.06	−0.35	−3.39 to 1.43		−0.71 ± 0.69	−0.75	−2.98 to 0.4	0.6191
	Mean		−0.55 ± 1	−0.35	−3.11 to 1.09		−0.66 ±0.63	−0.66	−3.02 to 0.28	0.5667

## Data Availability

The data that support the findings of this study are available from the corresponding author, upon reasonable request.
